# Effects of velocity on N_2_ and CO_2_ foam flow with in-situ capillary pressure measurements in a high-permeability homogeneous sandpack

**DOI:** 10.1038/s41598-023-36345-4

**Published:** 2023-06-20

**Authors:** Eric Vavra, Chutian Bai, Maura Puerto, Kun Ma, Khalid Mateen, George J. Hirasaki, Sibani Lisa Biswal

**Affiliations:** 1 Department of Chemical and Biomolecular Engineering, Rice University, 6100 Main Street, MS-362, Houston, TX 77005 USA; 2 TotalEnergies E&P R&T USA, 1201 Louisiana Street, Houston, TX 77002 USA

**Keywords:** Carbon capture and storage, Crude oil

## Abstract

The effects of velocity and gas type on foam flow through porous media have yet to be completely elucidated. Pressure drop and capillary pressure measurements were made at ambient conditions during a series of foam quality scan experiments in a homogenous sandpack while foam texture was simultaneously visualized. New insights into foam-flow behavior in porous media were discovered. Previously accepted “limiting” capillary pressure theory is challenged by the findings in this work, and the “limiting” terminology is replaced with the word “plateau” to reflect these novel observations. Plateau capillary pressure ${(P}_{c})$ and transition foam quality were found to increase with velocity. Transition foam quality was found to depend mostly on liquid velocity rather than gas velocity and is physically linked to foam type (continuous vs. discontinuous) and texture (fine vs. coarse). Distinct rheological behaviors also arose in the low- and high-quality foam regimes as a function of velocity. Foam flow was found to be strongly shear thinning in the low-quality regime where foam texture was fine and discontinuous. In the high-quality regime, the rheology was weakly shear thinning to Newtonian for coarsely textured foam and continuous-gas flow respectively. When all other variables were held constant, at ambient conditions, CO_2_ foam was found to be weaker with also lower capillary pressures than N_2_ foam and the differences in gas solubility is a likely explanation.

## Introduction

Foams have numerous promising applications in porous media ranging from enhanced oil recovery to CO_2_ storage in geologic formations^1–4^. A growing body of literature is dedicated to understanding the complexities of foam flow in porous media. The prediction of pressure gradient and liquid saturations for a given foam-flow system for a set of flow conditions is a general, yet elusive, goal in this field. The challenge lies in the complex interplay among relevant variables such as surfactant formulation, porous media type, and non-aqueous phase type.

Because the interactions among these variables are complex, fundamental studies are needed to deduce the true nature of foam in porous media and to identify it by simplifying relationships among the pertinent variables. One such study by Khatib and colleagues provided evidence for a “limiting” capillary pressure^5^. This theory greatly simplified foam models, as the capillary pressure could essentially remain a fixed variable. Another study by Hirasaki and Lawson laid the groundwork for predicting apparent viscosities of foams in porous media via a capillary tube model^6^. Falls et al. later extended these relationships to complex porous media with converging–diverging geometries^7^. In a separate work, Falls and colleagues clearly identified pertinent aspects of the shape and size of foam bubbles and related these features to mechanisms resulting in creation or destruction of bubbles^8^. Rossen identified a minimum pressure gradient required to mobilize foam^9,10^. Osterloh and Jante also clearly laid out the regimes in which foam flow becomes independent of either the gas or the liquid velocity^11^. Kam and Rossen identified the multiple steady states of certain foams^12^. Other works have explored the effects of relevant variables such as surfactant type and concentration^13–17^, salinity^17,18^, porous media heterogeneity^19^, porous media permeability^20,21^, and gas type^22–24^. These studies and others have significantly advanced understanding of foam flow in porous media, but predictive modeling capabilities have yet to be achieved^25,26^.

The aim of the present study is to further improve understanding of foam flow in porous media by examining fundamental relationships among capillary pressure, pressure gradient, apparent viscosity, and foam texture in a simple foam flow system that can be compared with several others from the literature^5,11,16,27,28^. To accomplish this, a large body of data was collected during foam quality scan experiments conducted at fixed gas velocities, in the style of Khatib et al.^5^, during which pre-generated foam was injected into a high-permeability sandpack and capillary pressure was measured via probe while in-situ observations of the foam were made.

## Materials and methods

### Chemicals and porous media

The surfactant selected for the study was Alpha olefin sulfonate AOS_14-16_ (activity = 39.03 wt%, Lot#7653919, Stepan®). A fresh solution of 1 wt% AOS_14–16_ was prepared with 3 wt% NaCl in Milli-Q® ultrapure water for each foam-flow test. Dry N_2_ gas (Airgas, 99.999% purity) and Bone-Dry CO_2_ gas (Airgas, 99.999% purity) were supplied by Matheson.

Properties of the sand pack and foam pre-generators for foam-flooding system testing are summarized in Table 1. The main body of the sand pack was constructed from a transparent polycarbonate tube with grooved rubber stoppers compressed into both ends. Rubber O-rings and two metal screens contained the sand in the tube. A metal compression cage prevented fluid leaks around the stoppers and ensured the sand remained in place. A capillary pressure probe was installed half-way down the length of the sand pack and was sealed into the main body by a rubber stopper with a silicone-gasket plug. The overall pressure drop across the sand pack was measured with a differential pressure transducer (Validyne). Further details of the pack construction are given in Vavra et al.^29^.

**Table 1:** Sand pack and foam pre-generator properties.

Foam pre-generator		Sand pack	
Property	Value	Property	Value
Sand mesh	20/40 (425–850 microns)	Permeability	143 Darcy
Length	1.2 in (3.0 cm)	Porosity	0.35
Outer Diameter	0.5 in (1.3 cm)	Sand mesh	20/40 (425–850 microns)
Body	Swagelok® F-series	Length	11.2 in (28.5 cm)
Body material	Stainless steel	Internal Diameter	2.0 in (5.08 cm)
		PV	210 cm^3^
		Body material	Polycarbonate

### Foam quality scan experimental procedure at fixed gas flow rate

#### Foam quality scan protocol

For all experiments, foam quality scans were conducted with constant gas-volumetric flow rates defined at standard conditions. Liquid flow rates varied to change the foam quality given by:

1 $${f}_{g}= {q}_{g}/({q}_{g}+{q}_{l})$$

where ${f}_{g}$ is foam quality, ${q}_{g}$[cm^3^/s] is evaluated at average-pressure and solubility-compensated gas volumetric flow rate at the midpoint of the pack, and ${q}_{l}$ [cm^3^/s] is liquid volumetric flow rate. The outlet of the sand pack was assumed to be at atmospheric pressure. The pressure-compensated flow rate, ${q}_{g}^{pressure-compensated}$, was calculated at the mid-length of the pack, where the capillary pressure probe was located, with the following formula:

2 $${q}_{g}^{pressure-compensated}= \frac{{(q}_{g}^{inlet}+{q}_{g}^{outlet})}{2}=\frac{{\big(q}_{g}^{outlet}\big(\frac{{P}^{outlet}}{{P}^{outlet}+\Delta P}+1 \big)\big)}{2}$$

where ${q}_{g}^{inlet}$ [cm^3^/s] and ${q}_{g}^{outlet}$ [cm^3^/s] are the volumetric gas flow rates at the inlet and outlet of the sand pack respectively, ${P}^{outlet}$ [psi] is atmospheric pressure at laboratory conditions, and $\Delta P$ [psi] is the pressure drop across the sand pack at steady-state. The solubility of CO_2_ for relevant average pressures was estimated and flow rates were corrected appropriately.

The gas and the aqueous phases were co-injected through a foam pre-generator before entering the sand pack, and the surfactant solution was recycled for each test. All tests were conducted from high-to-low foam quality. N_2_ gas was injected with flow rates defined at standard conditions of 1 cc/min, 3 cc/min, 9 cc/min, and 18 cc/min corresponding to standard superficial velocities of 2.3 ft/day (8.2 × 10^–6^ m/s), 7 ft/day (2.5 × 10^–5^ m/s), 21 ft/day (7.4 × 10^–5^ m/s), and 42 ft/day (14.8 × 10^–5^ m/s) respectively. CO_2_ gas was injected at standard conditions with a flow rate of 11.4 cc/min corresponding to a standard superficial velocity of 26.6 ft/day (9.4 × 10^–5^ m/s). A capillary pressure probe was sealed into the sand pack 5.6 in (14.25 cm) from the inlet. Details of the probe construction and static validation are provided in Vavra et al.^29^ Specifics of experimental conditions are given in Table 2.

**Table 2:** Summary of experiment conditions.

Flow rates	
Standard gas flow rate multiple and gas type	Pressure- and solubility-compensated total flow rate (gas plus liquid) range ft/day (m/s)
1 × N_2_	2.4–11.2 (8.2 × 10^–6^–4.0 × 10^–5^)
3 × N_2_	7.1–25.1 (2.5 × 10^–5^–8.9 × 10^–5^)
9 × N_2_	16.1–43.7 (5.7 × 10^–5^–15.4 × 10^–5^)
11.4 × CO_2_	22.4–47.3 (7.9 × 10^–5^–16.7 × 10^–5^)
18 × N_2_	32.9–54.8 (11.6 × 10^–5^–19.3 × 10^–5^)

Surfactant solution	
Property	Value
Surfactant type	AOS_14–16_
Surfactant concentration	1 wt%
NaCl content	3 wt%
Surface tension with N_2_	34 mN/m
Surface tension with CO_2_	33 mN/m

#### Pressure measurement and apparent viscosity

Capillary pressure and overall pressure drop were recorded by software every 5 s and averaged over a minimum of 0.25 PV of total injection at each flow condition. The temporal standard deviation was calculated. The apparent viscosity ${\mu }_{app}$ was then calculated from Darcy’s law assuming pseudo-single-phase flow:

3 $${\mu }_{app}=kA/({q}_{g}+{q}_{l})(\frac{\left|\Delta P\right|}{L})$$

where ${\mu }_{app}$ [cP] is apparent viscosity, $k$ [D] is absolute permeability, A [cm^2^] is the cross-sectional area of the sand pack, $\Delta P$ [atm] is the overall pressure drop, and $L$ [cm] is the length of the sand pack.

#### In-situ foam texture visualization

The texture of the foam flowing was filmed through the transparent wall of the sand-pack holder through a microscope objective. The visualized location was at the midpoint of the pack at the same distance from the inlet where the probe was located. The “footprint” of the foam directly contacting the wall was filmed at a resolution of 3080 × 1260 at 60 fps and 1290 × 1080 at 240 fps. LED ring lights were placed on the top, bottom, and side of the pack. This lighting configuration allowed for reflections in the plateau borders, where the foam contacted the inside wall of the polycarbonate sand-pack holder, to be visualized. The permeability at the wall of the pack is somewhat higher than that deeper into its center.

## Results and discussion

### Effect of gas flow rate on foam flow: capillary pressure, pressure gradient, apparent viscosity, and transition foam quality

#### Capillary pressure in-situ foam observations

**Figure 1:**
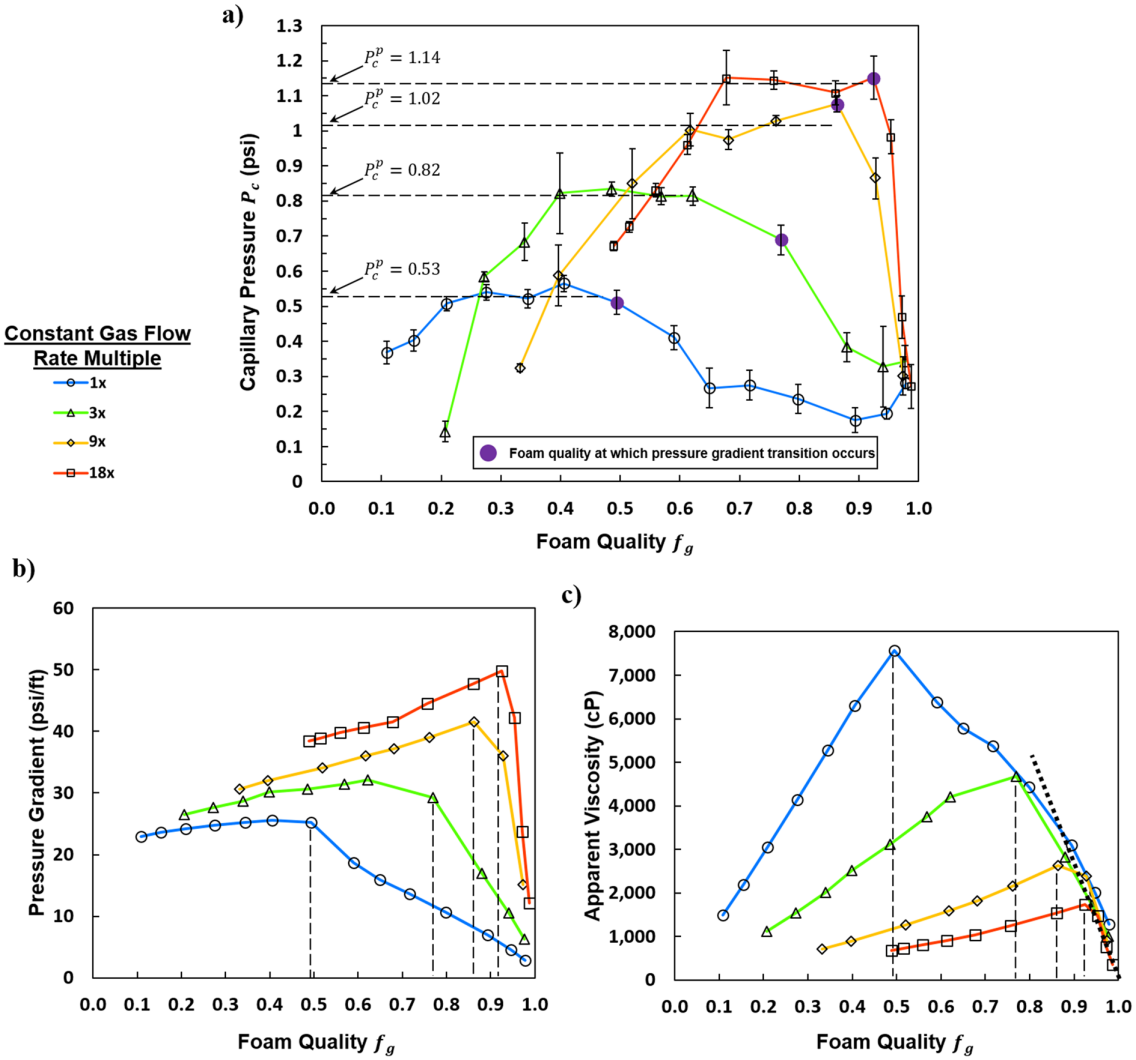
(**a**) Capillary pressure vs. foam quality. Foam quality transitions related to pressure gradient are marked with a purple point. Plateau capillary pressures are indicated with a horizontal dashed line. (**b**) Pressure gradient vs. foam quality. (**c**) Apparent viscosity vs. foam quality. The sloping dashed line represents the common behavior at high foam qualities. Vertical dashed lines indicate transition foam qualities. All experiments were conducted at constant standard gas flow rates with changing liquid (and therefore total flow rates) to obtain the various foam qualities. The colored lines are to guide the eye and represent a given gas flow rate.

The trends presented in Fig. 1 partially concur with those found in the literature, but some findings are different from several widely accepted assumptions of foam flow in porous media. Vavra et al. measured capillary pressure and found it to plateau over a range of foam qualities at a fixed gas velocity^29^. This finding is seemingly in agreement with Khatib et al. who were the first to measure a plateau in capillary pressure^5^. They identified the plateau as a “limiting” capillary pressure where the number of lamellae existing in the sand pack was only controlled by coalescence rate while the effect of generation rate was unmentioned. One aspect of evidence given in favor of this assumption by Khatib et al. was that the “limiting” capillary pressure was observed to decrease from 0.55 psi to 0.47 psi with a 2.3 × increase in gas rate^5^. It was argued that greater mechanical stresses, associated with increasing the gas velocity, resulted in more lamella rupture and thus a lower limiting value for capillary pressure^5^. Jimenez and Radke also showed that moving lamella rupture easier than when stationary^30^. Later, Farajzadeh et al. stated “as the lamella velocity increases, the limiting capillary pressure must decrease to compensate for the increase in disjoining pressure as the lamella stretches in passing from a pore throat to a pore body”^20^. On the other hand, in Fig. 1a, the plateau value for capillary pressure, indicated by a horizontal dashed line, increases from 0.53 psi to 0.82, 1.02, and 1.14 psi with increases in gas flow rate with multiples of 3 ×, 9 ×, and 18 × over the initial 1 × flow rate respectively. This finding suggests, for the tested conditions, similar to the conditions tested by Khatib et al.^5^, the number of lamellae in the foam at the plateau value is not only a function of coalescence by lamellae rupture, but also a function of other foam creation and/or destruction mechanisms such as lamella division and Ostwald ripening. For example, as gas velocity increases, the rate of lamella rupture could be expected to increase for the aforementioned reasons; however, the rate of foam generation by lamella division might also increase. Hence the term “plateau” is chosen to describe the flattened portion of the capillary pressure curves in Fig. 1a instead of “limiting” implying coalescence by lamella rupture as the dominating mechanism preventing further increase in capillary pressure. Alvarez predicted the “limiting” capillary pressure to increase with liquid velocity^31^. A similar observation was made by Ouali et al. who estimated capillary pressure from gas saturations measured by CT and found the maximum value, referred to as “${P}_{c}^{*}$” despite the lack of a plateau, to increase with increasing total velocity^32^. In their work, an appreciable plateau in liquid saturation (or capillary pressure) over a substantial range of foam quality was absent, but a peak value was observed much like that found by Kibodeaux and Rossen as well as Føyen et al. for some conditions^1,27,32^.

Another aspect of this study in agreement with Ouali et al. was measured capillary pressure decreasing with increasing foam quality in the high-quality regime^32^. For all tested gas rates, the capillary pressure “collapses” above the transition foam quality and converges on the same value of approximately 0.3 psi. The decrease in capillary pressure was observed regardless of velocity implying that, at some transition foam quality, the capillary pressure will always decrease. This process corresponded with foam coarsening, and might occur at an extremely dry foam quality for some foaming systems depending on surfactant formulation, porous media, gas type, etc. This decrease in capillary pressure above ${f}_{g}$ = 0.98 was also observed by Kibodeaux and Rossen for a constant gas rate experiment despite their experiments being with a rock core rather than a sand pack^27^. Ouali et al. and Føyen et al. observed the decrease in capillary pressure at foam qualities around ${f}_{g}$ = 0.8.^1,32^

**Figure 2:**
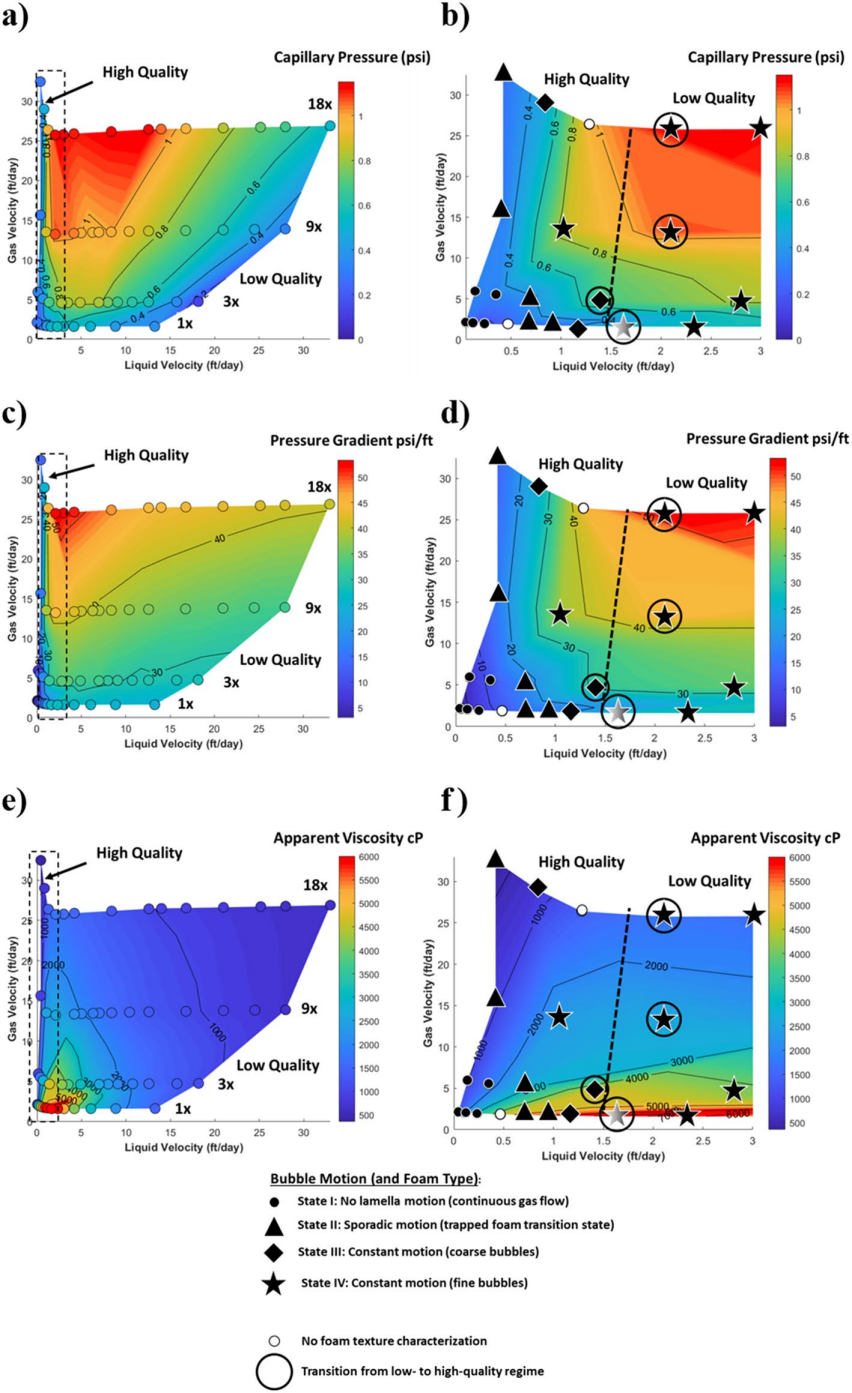
Osterloh- and Jante-style contour plots for N_2_ foam at reference (1 ×), 3 ×, 9 ×, and 18 × flow rates for equal axes ranges (left column) and shorter liquid velocity range (right column) of (**a**, **b**) Capillary pressure (**c**, **d**) Pressure gradient (**e**, **f**) Log of apparent viscosity. Circles in the left column of plots indicate where data was collected. Colors indicate magnitude. In the right column of plots, foam type and/or texture is indicated by the type of marker, and a nearly vertical, black-dashed trend line is drawn intuitively separating low- and high- quality regimes.

Experiments were conducted at a constant gas rate, but capillary pressure can be visualized in the style of Osterloh and Jante (as a function of the liquid and gas rate) in the contour plots of Fig. 2a and b^11^ (see Supplemental Video S1). In these plots, the magnitudes of capillary pressures correspond to colors. The points, indicated by circles or shapes, correspond to measurements made with the capillary pressure probe while all other information has been linearly interpolated. Figure 2a is to help in the visualization of capillary pressures for the entire variable space that was measured, and the roughly horizontal rows of points correspond to the various gas rates of the experiments (1 ×, 3 ×, 9 ×, and 18 ×). Figure 2b is plotted with a truncated liquid-rate axis, as found in the literature^11,16,31^, to better visualize the high-quality regime. Plotted in this way, for slow enough liquid velocities (less than approximately 10 ft/day), capillary pressure is relatively independent of the liquid rate in the low-quality regime where the plateau range occurs and, for fast enough gas velocities (greater than approximately 10 ft/day), relatively independent of the gas rate in the high-quality regime. The symbols indicate the foam type and texture that was observed in situ.

#### In-situ observations of foam texture and type

Foam texture (coarse vs. fine) and type (continuous vs. discontinuous) have been recognized as playing an important role in foam-flow behavior in porous media^5,8,31,33,34^. The “strongest” foams in terms of largest pressure gradients, lowest gas mobility, and highest capillary pressures are associated with fine (small bubble size) discontinuous (bubbles separated by lamellae) foams. “Weak” foams are either coarse (large bubble size) discontinuous foams or continuous-gas flow in which continuous gas channels flow through pockets of trapped discontinuous-gas foam pockets. Texture refers to bubble size and type refers to continuous vs. discontinuous gas. Fine foams exist in the low-quality regime. It has also been recognized that foam coarsens in the high-quality regime and becomes coarser with increasing foam quality^35^. Eventually, at dry enough foam qualities, the bubble size becomes on the order of the length scale of the porous medium, and a continuous-gas foam form. Note that the injection condition is not necessarily reflected in the saturation of the porous medium. In other words, a change in the fractional flow does not mean that there will be a corresponding change in the gas saturation. Fine foams at relatively low foam qualities lead to low gas mobility and low liquid saturations whereas coarse or continuous-gas flow at higher qualities is associated with higher gas mobilities and higher liquid saturations (Fig. 1a). In this study, foam flow states were categorized based on qualitative observations of the microscopy footage taken during foam-quality scan tests (see Supplemental Video S1). Foam flow was broadly categorized into four states given in the order in which the experiment was conducted:

1. *State I* For the driest and slowest injection conditions, lamellae were essentially stationary during the time over which they were observed. This likely means that most of the gas was flowing as a continuous phase, and any foam that was present was trapped. This condition occurred in the high-quality regime during the 1 × and 3 × flow rate experiments.
2. *State II* This state is similar to State I, but as injection conditions became wetter and liquid velocity increased, a steady-state “transition” foam type was observed where periodic lamellae movement occurred but was unsustained. This condition occurred in the high-quality regime and may be associated with periodic switching between continuous and discontinuous gas flow where most of the foam remains trapped.
3. *State III* Another transition foam type occurred in the high-quality regime that involved flowing coarse foam. This foam flow state occurred over a narrow range of conditions and corresponds with a sharp transition in ${P}_{c}$
4. *State IV* In the low-quality regime, fine-foam flow was observed.

The in-situ observations, of these foam-flow states, help in explaining the measured trends in capillary pressure, pressure gradient, apparent viscosity as a function of foam quality, liquid velocity, and gas velocity.

#### Pressure gradient

Pressure gradient curves are given in Fig. 1b for each gas rate evaluated. Each curve is divided into two distinct slopes as marked by a vertical dashed line. The low-quality regime, at foam qualities below the transition foam quality, and the high-quality regime at foam qualities greater than the transition foam quality. The low- and high-quality regimes are analogous to the liquid- and gas-rate independent regimes respectively that were first identified by Osterloh and Jante^11^. The pressure gradient trend is relatively independent of foam quality in the low-quality regime because the liquid flow rate was changed while the mass flow for the gas flow rate was held constant. Overall, there is a slight positive dependence of pressure gradient on liquid flow rate in the low-quality regime as seen in Fig. 2c. Small changes in gas compressibility with changes in pressure drop could contribute to the slightly positive slope in pressure gradients in this regime. In the high-quality regime in Fig. 1b, pressure gradients decline with foam quality with the steepness of the decline growing sharper with increasing gas rate. As indicated by the symbols in Fig. 2d, this decline in pressure gradients is associated with a transition from a fine foam to a continuous-gas foam with various steady states of coarse foam in between.

#### Apparent viscosity as a function of gas velocity

For decreasing the liquid rate at a given gas velocity, the apparent viscosity first increased up to a peak before decreasing with increasing foam quality (Fig. 1c). This trend appears identical to that for experiments conducted with total flow velocity fixed^36–38^. This is confirmed by examining Fig. 2e and following the trend in apparent viscosity for a line of either fixed gas velocity (zero slope) or fixed total velocity (slope = − 1). The relatively high apparent viscosities reported here are in accord with the presence of a “strong” foam and the high permeability of the porous medium. The transition foam quality separating the high- and low-quality regimes in apparent viscosity always corresponded to that for the pressure gradient despite changing liquid velocities. In the low-quality regime, changes in the liquid velocity have minimal impact on the apparent viscosity. Conversely, in the high-quality regime, small changes in the liquid velocity led to large changes in apparent viscosity (Fig. 2f).

In the low-quality regime the apparent viscosity decreases with increasing gas velocity due to shear thinning (Fig. 1c). This occurs despite an increase in the plateau capillary pressure with gas velocity (Fig. 1a). Therefore, foam types at the capillary-pressure plateau are not necessarily synonymous with “strong” foam, in terms of high-apparent viscosity. Another way to conceptualize foam strength is in terms of gas relative permeability. The same foam flow condition can be “stronger” in terms of relative gas permeability (i.e. lower gas permeability leads to lower liquid saturation and higher capillary pressures) but simultaneously “weaker” in terms of relative apparent viscosity. In actuality, the foam flow conditions exhibiting higher capillary pressures are stronger. Also of note, in the high-quality regime, where foam states I, II, and III are present (Fig. 2b,d,f), the apparent viscosity curves overlap across all tested gas rates (Fig. 1c). The overlapping region of the curves in the high-quality regime is a linear function of fractional flow as indicated by the diagonal dashed line in Fig. 1c.

#### Transition foam quality

Transition foam quality has been assumed by some to be independent of total velocity^16,39^. This assumption is critical for modeling foam flow in porous media and for predicting appropriate injection conditions into a reservoir. In some studies, test results support this assumption^5,40^. In others, the invariance of transition foam quality with increasing velocity is less clear^16,32,39^. Osterloh and Jante identified the transition foam quality by eye as the line of constant foam quality roughly separating the gas-rate-independent high-quality regime from the liquid-rate-independent low-quality regime^11^. This protocol has been followed by others^31^. In the present study, the transition between low- and high-quality regimes was identified based on the change in slope of the pressure gradient trends and was found to depend on the flow rate (Fig. 1b,c). This assertion is evident when drawing a line connecting the transition points shown as a dashed-black line on Fig. 2b, d, and f. The lines failed to follow a constant foam quality (a straight line passing through the origin). The transition from the low-to-high- quality regime corresponds to the transition from a fine-to-coarse foam texture. Thus, the transition foam quality is found to be physically linked to flow-rate-dependent mechanisms of foam that control foam texture. The transition foam quality generally depends more on changes in the liquid velocity than those in the gas velocity. The findings of Kahrobaei et al. generally agree with this assessment in that, the transition between distinct rheological regimes in the foam was a function of only the liquid velocity^16^. One hypothesis is that, at low gas rates, most of the gas is flowing continuously (and/or as a coarse foam). With increasing gas flow rate, more stationary bubbles are mobilized and foam generation by lamella or bubble division occurs resulting in a higher transition foam quality. Other factors might be playing a role. The fraction of bubbles that are stationary at any given point in time was observed to decrease with increasing gas velocity at constant foam quality. Also, the rate of bubble coarsening by Ostwald ripening relative to the average rate of bubble translation could be significant.

### Foam rheology

**Figure 3:**
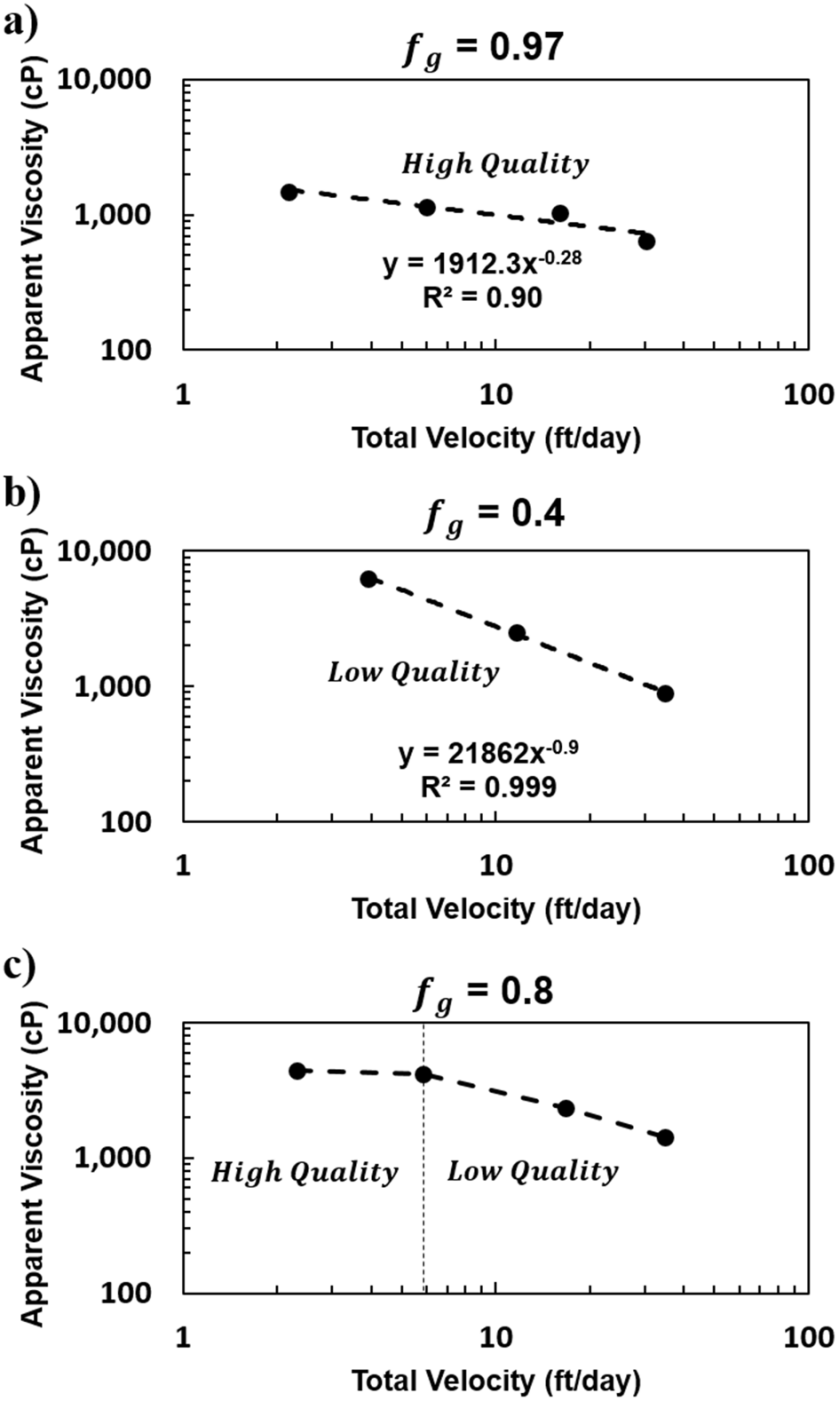
Log–log plot of apparent viscosity versus total superficial velocity at (**a**) ${f}_{g}$ = 0.97, (**b**) ${f}_{g}$ = 0.4, and (**c**) ${f}_{g}$ = 0.8 for N_2_ foam.

In assessing the rheology of foam flow in porous media, a log–log plot of apparent viscosity versus either total or gas superficial/interstitial velocity is commonly constructed for values of constant foam quality^6,7,31,32,40^. This plot has been constructed in Fig. 3 for both the high-quality and low-quality regimes across all flow rate experiments by interpolating the test results in Fig. 1c. The slope was found to depend on the flow regime of the foam. This dichotomy has been observed by others, and it is linked to the texture and type of foam flow^31,40^. A slope of − 0.28 was identified in the high-quality regime for ${f}_{g}$ = 0.97 (Fig. 3a). A slope of 0.00 would correspond to a Newtonian fluid. Because the foam in the high-quality regime is flowing as a coarse foam, the number of lamellae per unit length is relatively small, and the influence of these lamellae on apparent viscosity diminishes with decreasing number. As the foam transitions to a completely continuous-gas foam with further increases in ${f}_{g}$, the rheology behavior should become Newtonian. In the low-quality regime, where foam is fine, lamellae heavily influence the rheology. A slope of − 0.9 was identified for ${f}_{g}$ = 0.4 (Fig. 3b). This slope is larger in magnitude than the slope of − 0.67 reported by Falls et al. for bead-pack-generated foam in a glass bead pack, by Hirasaki and Lawson^6,7^ for “bulk” foam in smooth glass capillaries, and by Pedroni and Nabzar for in-situ generated foam in Fontainebleau sandstone cores^40^. A slope of − 1.0 would correspond to a perfectly shear-thinning fluid and has been observed by Persoff et al. in a Boise Sandstone^41^. Because the overall pressure drop was measured and a foam pre-generator was incorporated in the setup reported here, it is possible that end effects are affecting the measured pressure drops. Sand of the same type as that in the main pack was selected for the foam pre-generator to mitigate end effects potentially caused by injected gas slugs being either too small or too large. Indeed, transitions in pressure-drop trends coincided with capillary pressure trends, so end effects should be insignificant. Some researchers have concluded that foam texture will quickly adjust to the final form within a short distance from the entrance of the porous medium^42^. In-situ observations of foam texture appeared to show that this was also the case for the systems tested here by comparing visuals of the foam near inlet, middle, and outlet of the sandpack.

In Fig. 3c, upon transitioning between the low- and high-quality regimes, with increasing velocity, a distinct shift in slope is observed. This observation may be important in correctly interpreting apparent-viscosity results in that transition between the low- and high-quality foam regimes and could cause misinterpretation of the foam rheology if unrecognized.

### Comparison of nitrogen and carbon dioxide foam

**Figure 4:**
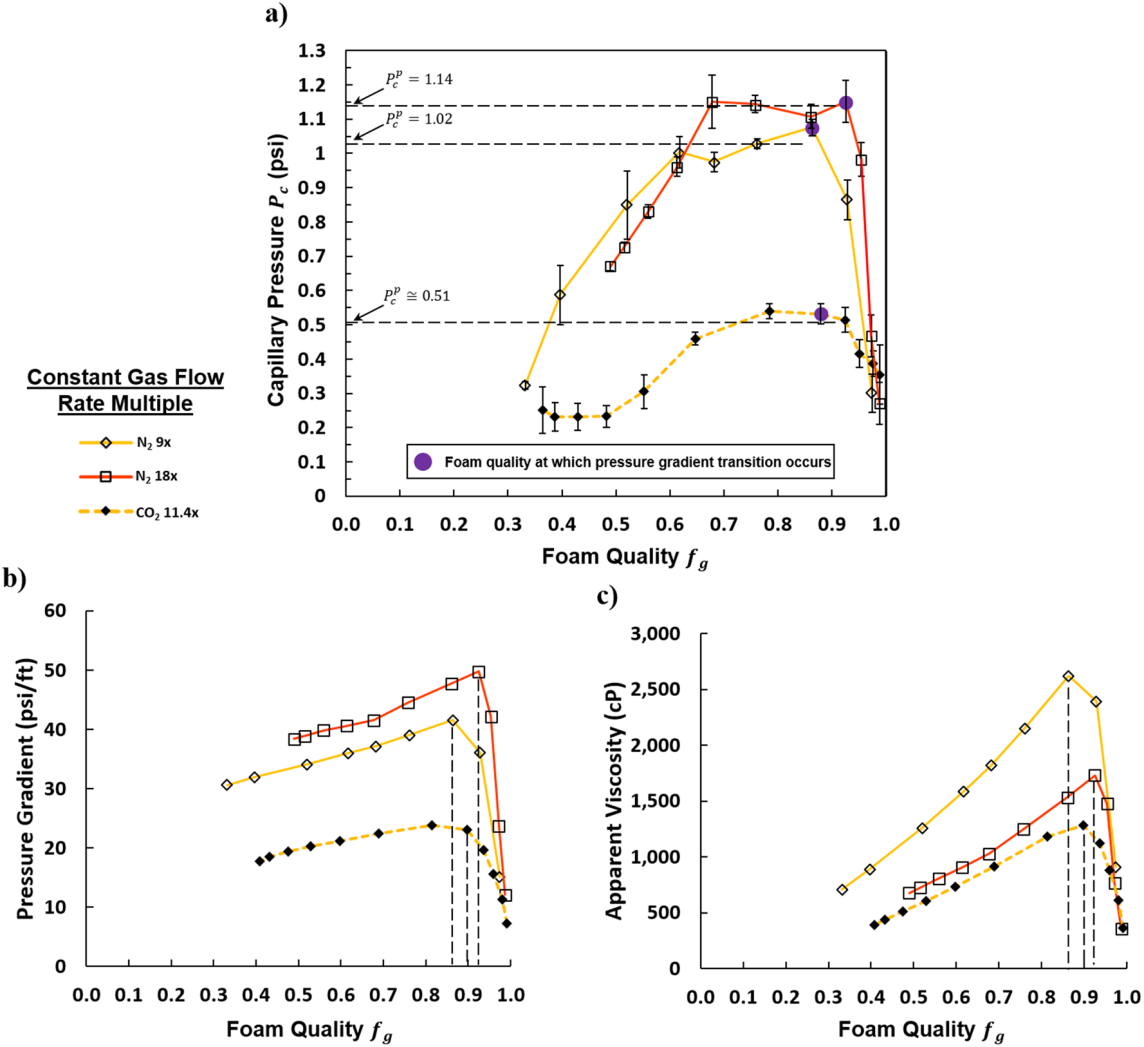
(**a**) Capillary pressure vs. foam quality. Foam quality transitions related to pressure gradient are marked in purple. Plateau capillary pressures are indicated with a horizontal dashed line. (**b**) Pressure gradient vs. foam quality. (**c**) Apparent viscosity vs. foam quality. vertical dashed lines indicate transition foam qualities. All quantities were measured at constant gas rates (CO_2_ foam at 11.4 × flow rate and N_2_ foam at 9 ×, and 18 × flow rates). The colored lines are to guide the eye and represent a given gas flow rate.

As seen in Fig. 4a, the measured capillary pressures for CO_2_ foam are lower than those of the N_2_ cases at similar flow rates across all conditions. This finding might be expected because CO_2_ foam is generally recognized as being “weaker” than N_2_ foams^22–24,43,44^, and a higher gas mobility, associated with “weaker” foams, would lead to a high liquid-phase saturation and consequently a lower capillary pressure. The plateau in ${P}_{c}$ for the CO_2_ case appears to be more rounded, and it contains fewer data points than those of the N_2_ cases.

At the test conditions, the surface tensions of each system are nearly identical. Despite this, CO_2_ exhibits similar transition foam quality to N_2_ (Fig. 4b), but CO_2_ foam also resulted in lower pressure gradients. This might indicate that the foam generation is independent of gas type; however, due to the high solubility of CO_2_ in the aqueous phase, Ostwald ripening/diffusion coarsening is playing a more significant role in CO_2_ foam destruction. Zeng et al. found that lamella permeability to gas is predicted to be the primary driver of differences in foam strength (in terms of pressure gradient or apparent viscosity)^22^. Higher film permeability leads to faster foam destruction by diffusion coarsening, and film permeability is proportional to gas solubility. If Ostwald ripening is the dominant mechanism of foam destruction, then the conventional accepted limiting capillary pressure theory does not accurately describe the foam behavior. This is because lamella rupture, at the limiting capillary pressure, is the only mechanism of bubble destruction considered to prevent foam texture from becoming finer in the established theory. However, it has also been argued that diffusion coarsening plays a small role in foam destruction in porous media under certain conditions, so more experiments are needed to explore if Ostwald ripening is the factor driving the differences among gas types^45^.

The transition foam qualities are indicated by the vertical dashed lines on Fig. 4b and c. For the CO_2_ 11.4 × flow rate experiment, the transition foam quality occurs around ${f}_{g}$ = 0.93, which is very close to the same value for the comparable N_2_ case (${f}_{g}$ = 0.92) and similar to literature reference values of 0.94 from Osterloh and Jante or 0.97 from Khatib et al.^5,11^. Corresponding to the lower capillary pressures for CO_2_ foam in Fig. 4a, lower pressure gradients and apparent viscosities are also observed.

## Conclusion

Foam quality scans were conducted at fixed gas velocity in a 143-Darcy sand pack with foam pre-generation. A known strong-foaming solution of 1 wt% AOS_12-16_ in 3 wt% NaCl brine was selected. The overall pressure drop and capillary pressure were measured, and the microscopic foam structure was filmed in-situ. Both N_2_ and CO_2_ gas foams were tested. The following insights were gained:

1. A plateau capillary pressure occurs in the low-quality regime across all velocities and test conditions evaluated in these experiments. The plateau values measured for capillary pressure in the N_2_ system were 0.53, 0.82, 1.02, and 1.14 psi for standard superficial gas velocities of 2.3 ft/day (8.2 × 10^–6^ m/s), 7 ft/day (2.5 × 10^–5^ m/s), 21 ft/day (7.4 × 10^–5^ m/s), and 42 ft/day (14.8 × 10^–5^ m/s) respectively.
2. The capillary pressure curves of different gas velocities for the N_2_ foam system decrease in the high-quality regime with increasing foam quality to a convergent value of 0.3 psi.
3. Transitions in capillary pressure and pressure gradient behavior were linked to foam texture (fine vs. coarse) and flow type (discontinuous vs. continuous). Fine foam in the low-quality regime exhibits high pressure gradients and contains a region of plateau capillary pressure. Pressure gradient and capillary pressure for coarse foams or continuous gas flow in the high-quality regime are a strong function of the gas fractional flow and liquid velocity.
4. Transition foam qualities correspond to a transition between a coarse and a fine foam and depend mainly on the liquid velocity.
5. Apparent viscosity curves of different gas velocity collapse onto one another in the high-quality regime and are a linear function of the fractional flow.
6. Foam rheology is significantly shear thinning in the low-quality regime with a power-law exponent of − 0.9 and is closer to Newtonian in the high-quality regime with an exponent of − 0.28. Transitioning between regimes with increases in gas velocity resulted in a shift in the rheological trend for the same foam quality.
7. The CO_2_ foam expectedly exhibited lower pressure gradients and apparent viscosities than N_2_ foam while both gas types resulted in similar transition foam quality. Plateau capillary pressure for the CO_2_ foam was also lower than that of the N_2_ foam in accord with the lower pressure gradients. The differences between gas types might be due to differences in the permeability of lamellae in the foam to each gas.

Some implications of these conclusions are as follows:

1. In at least some systems in which foam is flowing in porous media, conventional “limiting” capillary pressure theory fails to explain the capillary pressure curve.
2. Because the plateau capillary pressure was measured to be a function of velocity in this work, future studies will likely need to examine this relationship in detail to mathematically describe the relationship between velocity and the capillary pressure curve for foam flow in porous media.
3. Foam rheology is dependent on foam type (continuous vs. discontinuous gas) and texture (fine vs. coarse) and, by extension, the foam flow regime (high vs. low quality). Therefore, foam rheology is nuanced and cannot be described unless the foam texture is known.
4. For foams with highly soluble gas phases, Ostwald ripening should be given more consideration as a foam destruction mechanism. This is not considered in conventional limiting capillary pressure theory.

## Supplementary Information

Supplementary Video 1.
